# Cucumber-Derived Exosome-like Vesicles and PlantCrystals for Improved Dermal Drug Delivery

**DOI:** 10.3390/pharmaceutics14030476

**Published:** 2022-02-22

**Authors:** Abraham M. Abraham, Sabrina Wiemann, Ghazala Ambreen, Jenny Zhou, Konrad Engelhardt, Jana Brüßler, Udo Bakowsky, Shu-Ming Li, Robert Mandic, Gabriella Pocsfalvi, Cornelia M. Keck

**Affiliations:** 1 Department of Pharmaceutics and Biopharmaceutics, Philipps-Universität Marburg, Robert-Koch-Str. 4, 35037 Marburg, Germany; abraham.abraham@pharmazie.uni-marburg.de (A.M.A.); sabrina.wiemann@pharmazie.uni-marburg.de (S.W.); ghazala.ambreen@pharmazie.uni-marburg.de (G.A.); konrad.engelhardt@pharmazie.uni-marburg.de (K.E.); jana.bruessler@staff.uni-marburg.de (J.B.); ubakowsky@aol.com (U.B.); 2 EVs & MS Research Group, Institute of Biosciences and BioResources (IBBR), National Research Council of Italy, (CNR), 80131 Napoli, Italy; gabriella.pocsfalvi@ibbr.cnr.it; 3 Department of Otorhinolaryngology, Head and Neck Surgery, Campus Marburg, University Hospital Giessen and Marburg, Baldingerstrasse, 35033 Marburg, Germany; mandic@med.uni-marburg.de; 4 Department of Pharmaceutical Biology and Biotechnology, Philipps-Universität Marburg, Robert-Koch-Str. 4, 35037 Marburg, Germany; jenny.zhou@pharmazie.uni-marburg.de (J.Z.); shuming.li@staff.uni-marburg.de (S.-M.L.)

**Keywords:** PlantCrystals, cucumber, cucumber juice derived exosome-like vesicles, extracellular vesicles (EVs), transdermal drug delivery, high pressure homogenization

## Abstract

(1) Background: Extracellular vesicles (EVs) are considered to be efficient nanocarriers for improved drug delivery and can be derived from mammalian or plant cells. Cucumber-derived EVs are not yet described in the literature. Therefore, the aim of this study was to produce and characterize cucumber-derived EVs and to investigate their suitability to improve the dermal penetration efficacy of a lipophilic active ingredient (AI) surrogate. (2) Methods: The EVs were obtained by classical EVs isolation methods and by high pressure homogenization (HPH). They were characterized regarding their physico-chemical and biopharmaceutical properties. (3) Results: Utilization of classical isolation and purification methods for EVs resulted in cucumber-derived EVs. Their dermal penetration efficacy for the AI surrogate was 2-fold higher when compared to a classical formulation and enabled a pronounced transdermal penetration into the viable dermis. HPH resulted in submicron sized particles composed of a mixture of disrupted plant cells. A successful isolation of pure EVs from this mixture was not possible with classical EVs isolation methods. The presence of EVs was, therefore, proven indirectly. For this, the lipophilic drug surrogate was admixed to the cucumber juice either prior to or after HPH. Admixing of the drug surrogate to the cucumber prior to the HPH resulted in a 1.5-fold increase in the dermal penetration efficacy, whereas the addition of the AI surrogate to the cucumber after HPH was not able to improve the penetration efficacy. (4) Conclusions: Results, therefore, indicate that HPH causes the formation of EVs in which AI can be incorporated. The formation of plant EVs by HPH was also indicated by zeta potential analysis.

## 1. Introduction

**Figure 1:**
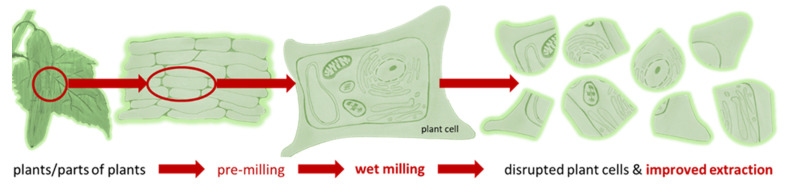
Scheme of PlantCrystal-technology. Plants or parts of plants are subjected to wet milling (bead milling or high-pressure homogenization) to destroy the plant cells exhaustively. This allows for an improved and solvent-free extraction of plant constituents when compared to classical extraction methods.

PlantCrystals are composed of plants and/or parts of plants and possess sizes <1 µm. They are produced in a similar way to drug nanocrystals, which are typically produced by bead milling or high pressure homogenization [1,2,3,4]. Therefore, the name “PlantCrystals” is a combination of the words “plants” and “nanocrystals”, i.e., it combines the source and the production method of the PlantCrystals. Wet milling enables an efficient destruction of plant cells, which typically possess sizes >10 µm. The destruction of the plant cells and organelles allows for a fast and exhaustive extraction of plant constituents without the need of organic solvents (Figure 1). Thus, the PlantCrystal-technology is considered to be a novel, environmentally friendly plant extraction process; the suitability of the technology over classical extraction methods was already proven in various recent studies [1,4,5,6,7,8,9,10,11,12]. These studies could, for example, show an enhanced extraction efficacy, especially for lipophilic compounds when compared to classical extraction methods and could also demonstrate an improved therapeutic efficacy when compared to classical extracts. The reason is the efficient destruction of the plant material during bead milling and/or high-pressure homogenization (HPH). HPH has been used for >100 years for the destruction of particles and is well known to cause cell rupture [13]. Recently, HPH was also shown to enable the formation of exosomes from a human glioblastoma cell line [14].

Exosomes are extracellular vesicles (EVs) and are similar to liposomes in terms of size, shape and structure, but have a more complex lipid bilayer, containing up to hundreds of different lipids and proteins, as well as internal cargo and surface-associated molecules [15]. Exosomes’ biogenesis is still unclear, but they are released by various cell types and are present in different biological fluids or in vitro cell culture supernatants [16,17,18]. Exosomes play an important and vital role in cell–cell communication [16,17,19], are able to deliver biomolecules into distant recipient cells and, thus, can be used as nanoscale drug delivery carriers [17,20,21].

More recent studies also reported the presence of plant exosome-like vesicles (PEVs) that are similar to mammalian and cell culture supernatant EVs in terms of shape, size, isolation and characterization methods [16,22,23,24,25,26,27,28,29]. So far, PEVs were already isolated from different plants. For example, PEVs were isolated as apoplastic vesicles from tomato leaves and sunflower seed and from several edible plants juices such as clementine, lemon, grapefruit, grape, carrot, watermelon, broccoli or ginger by using classical isolation methods for EVs [22,23,24,27,30,31,32,33] and their potential for improved drug delivery was already demonstrated for various routes of administration [29]. Nonetheless, the potential of PEVs to improve the penetration efficacy of active pharmaceutical ingredients for dermal application was only rarely demonstrated; more detailed investigations are needed in this regard [29,33]. In addition, a study that investigates whether HPH can form PEVs that allow for improved drug delivery is also not yet available. Therefore, the aim of this study was to produce PEVs by classical EVs isolation methods (classic PEVs) [29] and by HPH (PlantCrystal-PEVs) [1,34] and to investigate if PlantCrystal-PEVs are as effective for improved (dermal) drug delivery as classic PEVs. 

*Cucumis sativus L.* from the *Cucurbitaceae* family is very popular in many skin care products [35]; cucumber-derived PEVs were not yet isolated and investigated for dermal drug delivery. Therefore, cucumber was chosen for the isolation and production of classic PEVs and PlantCrystal-PEVs. The PEVs were characterized regarding their physico-chemical properties and the dermal penetration efficacy was determined ex-vivo on fresh porcine skin. DiI perchlorate (DiI) is a fluorescent dye and was used as surrogate for a lipophilic active ingredient (AI).

## 2. Materials and Methods

### 2.1. Materials

Ten randomly selected cucumbers (*Cucumis sativus* L.) were bought from a local supermarket in Marburg/Germany and the origin according to the supplier was Spain. The AI surrogate DiI (1,1′-dioctadecyl-3,3,3′,3′-tetramethylindo-carbocyanine perchlorate) was obtained from Biozol Diagnostica Vertrieb GmbH (Eching, Germany). The protease inhibitor cocktail contained 1 mol/L sodium azide (Sigma-Aldrich Chemie GmbH, Steinheim, Germany), 100 mmol/L PMSF (phenylmethylsulfonyl fluoride, Roche Diagnostics GmbH, Mannheim, Germany) and 1 mmol/L leupeptin (Roche Diagnostics GmbH, Mannheim, Germany). Phosphate buffered saline (PBS, pH 7.4 at 25 °C) composed of 100 mmol/L phosphate and 10 mmol/L ethylenediamine tetra acetic acid (EDTA) was used as extraction buffer. Purified and filtered (0.22 µm) water was freshly obtained from a PURELAB Flex 2 (ELGA LabWater, High Wycombe, UK).

### 2.2. Methods

#### 2.2.1. Preparation of Classic PEVs

Four randomly chosen cucumbers were weighed (total weight 936 g) and washed thrice with cold running tap water for 5 min. An additional washing step was performed by using purified water. The washed cucumbers were then left to air dry at room temperature, peeled using a ceramic knife and weighted again. The total weight after peeling was 629 g. The peeled cucumbers were cut into smaller pieces and juiced using a blender (Philips HR3655/00 Blender, Philips Electronics NV, Amsterdam, Netherlands). After adding the protease inhibitor cocktail, filtered (0.22 µm) PBS was added to reach a final volume of 1000 mL. Subsequently, the isolation of the PEVs was performed by differential ultracentrifugation (DUC) [16]. DUC included a series of low-velocity cycles at 400, 800, 2000 and 15,000× *g* using a Sorvall centrifuge (RC 6 Plus Centrifuge, Fisher Scientific GmbH, Schwerte, Germany) with a fixed angle rotor, each for 30 min at room temperature. The pellets were discarded; the resulting supernatants were collected. The supernatant after 15,000× *g* was filtered (0.45 µm) and centrifuged at 120,000× *g* for 60 min at 4 °C using a Sorvall ultracentrifuge (MTX 150 Micro-Ultracentrifuge, Fisher Scientific GmbH, Schwerte, Germany) with a S50-A fixed angle rotor. The supernatants were carefully discarded without disturbing the pellet. The last step was repeated, and fresh samples were added into the same tubes until all the starting quantity was ultracentrifuged. The final pellet obtained was expected to contain the cucumber-derived classical PEVs. It was resuspended in a small volume of PBS and vortexed vigorously for about 20 min. Samples obtained were stored at −80 °C until further use.

#### 2.2.2. Preparation of PlantCrystal-PEVs

Cucumber was washed, peeled and juiced as described in Section 2.2.1. The cucumber juice was then subjected to rotor-stator high-speed stirring (HSS, Ultra Turrax T25, IKA, Staufen, Germany) for 1 min at 5000 rpm (×2) and 1 min at 8000, 10,000 and 12,000 rpm, respectively. This pre-milling by HSS is an important step to destroy larger plant particles, which avoids blockage of the gap during piston-gap HPH. In the next step, HPH was applied by using a LAB 40 in discontinuous mode (GEA Niro Soavi, Lübeck, Germany). The first homogenization cycles were conducted at low pressure (3 × 200 bar); the second cycles were homogenized at medium pressure (3 × 500 bar, 3 × 750 bar, 6 × 1000 bar); the third step was the high-pressure homogenization (3 × 1500 bar).

#### 2.2.3. Purification of PEVs

Size exclusion chromatography (SEC) was used for the isolation of the PEVs from their crude suspensions to allow for a thorough physico-chemical characterization of the PEVs. SEC is considered a reproducible, low cost and non-destructive purification method for EVs [36,37,38]. SEC was performed with an qEV column from Izon Science (Izon Science, Medford, MA, USA). The column was washed with 30 mL filtered PBS (0.22 µm) before loading a volume containing 500 µg PEVs (expressed as protein quantity) onto the column; then, 1 mL of filtered (0.22 µm) PBS was added, eluted through the column and 500 µL were collected. This was repeated until thirty fractions were obtained. The PEVs yield after the purification was calculated using the following equation:

(1) $$yield\;\left(\%\right)=\left(\frac{\mathrm{amount}\;\mathrm{of}\;\mathrm{protein}\;\mathrm{obtained}\;\mathrm{after}\;\mathrm{purification}}{\mathrm{the}\;\mathrm{intial}\;\mathrm{amount}\;\mathrm{loaded}\;\mathrm{before}\;\mathrm{purification}}\right)\times 100$$

In addition to the above-described protocol, for the PlantCrystal-PEVs, after SEC, gradient ultracentrifugation (GU) was also applied for the purification of the PEVs. GU was performed using cushions composed of 1 and 2 mol/L sucrose prepared in Tris-HCl/D_2_O, and centrifuged at 110,000× *g* for 120 min at 4 °C using a SW 32 Ti rotor (Beckman Coulter, Brea, CA, USA) [22]. The purified PEVs were analysed regarding their physico-chemical properties (cf. Section 2.2.4).

#### 2.2.4. Physico-Chemical Characterization of PEVs

The crude PEVs and the purified PEVs were characterized regarding size, shape, zeta potential (ZP), protein content and protein profile. The size was determined by dynamic light scattering (DLS), nanoparticle tracking analysis (NTA) and laser diffractometry (LD). The shape and morphology of the formulations were determined by light microscopy (LM), scanning electron microscopy (SEM) and atomic force microscopy (AFM). The ZP was determined by laser Doppler anemometry (LDA); the protein content was analysed with the bicinchoninic acid (BCA) assay; the protein profile of the PEVs was determined by sodium dodecyl sulphate-polyacrylamide gel electrophoresis (SDS-PAGE). Further details of the methods used are given below.

##### Dynamic Light Scattering (DLS)

DLS (Zetasizer Nano ZS, Malvern-Panalytical, Kassel, Germany) was used to determine the size of the particles as hydrodynamic diameter (z-average) and the polydispersity index (PdI) as measure for the width of the size distribution [39]. Measurements were performed in triplicate; the measuring conditions were adjusted to 20 °C. The data were analysed with the general-purpose mode built in the software of the instrument. 

##### Nanoparticle Tracking Analysis (NTA)

The size distribution and the particle number/concentration of the PEVs were also analysed via NTA using a ZetaView PMX 420 (Particle Metrix, Meerbusch, Germany). Prior to the measurements, the crude PEVs were diluted 100,000 times and the purified samples were diluted 100 to 500 times in filtered PBS (0.2 µm) to obtain 20–60 vesicles per field of view for optimal tracking. Each measurement was performed by scanning 11 cell positions each and capturing in scatter mode 60 frames per position under the following settings: camera sensitivity: 80.0, shutter: 100. Three videos of 30 s were taken and analysed by using the in-build ZetaView 8.05.12. SP2 software. The area under the histogram for each sample was analysed in triplicate; the measurements were averaged and used as one particle concentration measurement. All NTA measurements were performed with identical system settings for consistency.

##### Laser Diffractometry (LD)

LD (Mastersizer 3000, Malvern-Panalytical, Kassel, Germany) was performed to detect possible large particles within the PlantCrystal-PEVs samples. Measurements (n = 3) were performed while stirring the sample at 1750 rpm without sonication. Mie-theory was used for the analysis of the data with optical parameters set to 1.52 (real refractive index) and 0.1 (imaginary refractive index) [39]. Results are expressed as median volume-based diameters (d(v)0.1–d(v)0.99).

##### Light Microscopy (LM)

LM was performed with an Olympus BX53 microscope (Olympus Corporation, Tokyo, Japan) equipped with a SC50 CMOS color camera (Olympus soft imaging solutions GmbH, Münster, Germany).

##### Scanning Electron Microscopy (SEM)

For SEM analysis diluted PEVs in PBS were negatively stained with 2% (w/w) uranyl acetate (Sigma-Aldrich Chemie GmbH, Steinheim, Germany). After 1 h, the samples were placed onto a sample holder and left at room temperature to dry for 3 h. The samples were then sputter-coated with platinum to increase conductivity and transferred into the SEM chamber. Images were acquired by using a JSM-7500F SEM (JEOL Germany, Munich, Germany) at a voltage of 5.0 kV [40].

##### Atomic Force Microscopy (AFM)

The samples were diluted (1:1000) with filtered (0.22 µm) PBS and were pipetted onto a silicon wafer (1 × 1 cm^2^). After 20 min of incubation, the suspension was removed by aspirating the extra water, leaving the tested samples on the silica wafer. AFM was then performed on a NanoWizard 3 NanoScience AFM (JPK Instruments, Berlin, Germany). The microscope was vibration-damped. Commercial 1-levertips (NSC 14 Al/BS) on a cantilever with a length of 125 µm and a resonance frequency of about 140 kHz and a force constant of 5 N/m were applied. Measurements were performed in tapping mode in the air. The scan speed was adjusted between 0.5 and 1.5 Hz [40].

##### Zeta Potential Analysis (ZP)

The ZP represents the electric potential at the slipping plane of the electrical double layer and is considered a vital parameter to predict the physical stability of nanocarriers. The ZP was assessed by measuring the electrophoretic mobility (EM) via LDA (Zetasizer Nano ZS, Malvern-Panalytical, Kassel, Germany). The EM was then converted into the ZP by using the Helmholtz–Smoluchowski equation. The measurements were performed in conductivity-adjusted purified water (50 μS/cm/20 °C) and/or the used extraction buffer [34,41]. The analysis was performed in triplicates and is shown as an average ± standard deviation (SD).

##### Protein Quantification

The protein amounts of the PEVs were quantified by using the BCA assay with micro BCA kits (Micro BCA Protein Assay Kit, Thermo Fisher Scientific, Rockford, IL, USA), following the protocol provided by the supplier. The absorbance values were obtained by using the NanoPhotometer^®^ NP80 system (IMPLEN, München, Germany).

##### Protein Profiling

The protein composition of the PEVs was determined by SDS-PAGE using the protocol of Mandic et al., and Bokka et al. [24,42]. For this, the gel was prepared using the previously described procedures [43] and PEVs (equal to 30 μg protein measured by BCA assay) were loaded onto the gel. The proteins were then electrophoretically separated in reducing conditions by using Bolt MOPS SDS running buffer (Invitrogen, Carlsbad, CA, USA). In a final step, the proteins were stained with colloidal Coomassie blue (Applichem GmbH, Darmstadt, Germany).

#### 2.2.5. Dermal Penetration Efficacy of PEVs

The dermal penetration efficacy of classical PEVs and PlantCrystal-PEVs was assessed in three steps. The first step included the preparation of the AI-surrogate-loaded PEVs. The second step was the penetration experiment utilizing the ex-vivo porcine ear response model and the third step was digital image analyses to obtain the objective penetration parameters, i.e., mean penetration depth (MPD) and the amount of penetrated AI-surrogate (APA). In addition, the stratum corneum thickness (SCT) and changes of the SCT upon the treatment with the different formulations were assessed. The SCT is a sensitive parameter to detect changes in skin hydration [44,45,46]; thus, by assessing changes in the SCT that occur upon the treatment with the PEVs, it was hypothesized to gain more detailed information on the underlying penetration mechanisms of the different PEVs.

##### Preparation of AI-Surrogate-Loaded PEVs

The classical PEVs were loaded by adding DiI (10 nM) to the crude classical PEVs. The obtained samples were gently vortexed for 15 min and then incubated for 30 min at room temperature in the dark. Ultracentrifugation was applied (cf. Section 2.2.1) to remove excess (i.e., free or aggregated crystalized) AI-surrogate that was not incorporated into the PEVs [47]. PlantCrystal-PEVs were produced by adding DiI (10 nM) to the cucumber juice and by processing the mixture with HSS and HPH, as described in Section 2.2.2. DiI (10 nM) in PBS (DiI-PBS) served as control. In addition, unloaded PlantCystal-PEVs and PlantCystal-PEVs to which DiI (10 nM) was added after HSS and HPH were also used as benchmark controls (Table 1).

##### Determination of Dermal Penetration Efficacy with the Ex-Vivo Porcine Ear Model

The dermal penetration efficacy was determined ex-vivo using porcine ears that were freshly obtained from a local slaughterhouse [48,49,50]. The ears were washed with lukewarm water (23–25 °C), dried with soft lint-free tissue and used directly on the day of slaughtering. Intact skin areas without visible scratches and wounds were selected and marked. The barrier integrity of the skin was ensured by measuring the transepidermal water loss (TEWL) with a Tewameter^®^ TM 300 (Courage+Khazaka electronic GmbH, Köln, Germany) and only skin areas that possessed TEWL-values ≤12 g/m^2^/h were included in the study. An amount of 12.5 µL of each sample was applied on an examination area of 1 × 1 cm^2^ on the dorsal side of the ear. The formulations were carefully distributed by using a saturated latex glove finger [51] and incubated at 32 °C for a penetration time of 6 h. Afterwards, punch biopsies (Ø 10 mm) were taken and immediately embedded using Tissue-Tek^®^ (O.C.T.™, Sakura Finetek Europe B.V., Alphen aan den Rijn, The Netherlands), frozen and stored at −20 °C. In the next step, the frozen skin biopsies were cryosectioned into 20 µm thick vertical cross-sections by using a cryomicrotome (Frigocut 2700, Reichert-Junk, Nußloch, Germany). The skin sections obtained were subjected to inverted epifluorescence microscopy (Olympus CKX53 equipped with an Olympus DP22 color camera, Olympus Deutschland GmbH, Hamburg, Germany). The exposure time was adjusted to 50 ms and the intensity of the fluorescent light source to 100% and kept constant for all images. The used fluorescence filter was the DAPI HC filter block system (excitation filter: 540–560 nm, dichroic mirror: 570 nm, emission filter: from 580 nm (LP)) and the magnification was 200-fold. Each formulation was tested in triplicate, i.e., on three different and independent ears. From each sample a total of at least 108 images, i.e., 36 images per skin biopsy and three skin biopsies for each sample were obtained, which were used for subsequent digital image analysis.

##### Digital Image Analysis

Digital analysis of the images was performed by using ImageJ software [52,53] and according to previously established procedures [44,45]. For this, all images were subjected to an automated threshold protocol to eliminate the auto-fluorescence of the skin (cf. Supplementary Section S1). The remaining light intensity of the images corresponded to the amount of penetrated AI (APA) and was determined as mean grey value/pixel (MGV/px). In addition, the mean penetration depth (MPD) of the AI surrogate and the stratum corneum thickness (SCT) were measured with the scale function of the software. The scale was set to 2.84 px/µm; the SCT was measured from the original images and the MPD was determined from the thresholded images, respectively. The values obtained were calculated as relative values. The relative APA and the relative MPD were calculated by setting the APA and the MPD of the DiI-PBS to 100%. The relative SCT was calculated by setting the SCT of the non-treated skin to 100%.

#### 2.2.6. Statistical Analysis

Descriptive statistics were calculated by using Microsoft Excel^®^ and are reported as mean ± standard deviation (SD). JASP software (version 0.13.1.0) [54] was used for the comparison of the mean values. Tests for normal distribution and variance homogeneity of the data were conducted using the Shapiro–Wilk test and the Levene’s test, respectively. For normally distributed data, mean values were compared by one-way ANOVA, utilizing a Welch-correction in case of variance heterogeneity. Adequate post-hoc tests (Tukey, Games–Howell) were performed to compare the mean values to each other. For non-parametric data sets, Kruskal–Wallis analysis of variance with Dunn’s post-hoc comparisons were performed. To account for alpha error accumulation Bonferroni–Holm correction was used. *p*-values <0.05 were considered statistically significant.

## 3. Results and Discussion

### 3.1. Cucumber-Derived PEVs Produced by Classical Methods

#### 3.1.1. Characterization of Crude Classical PEVs

**Figure 2:**
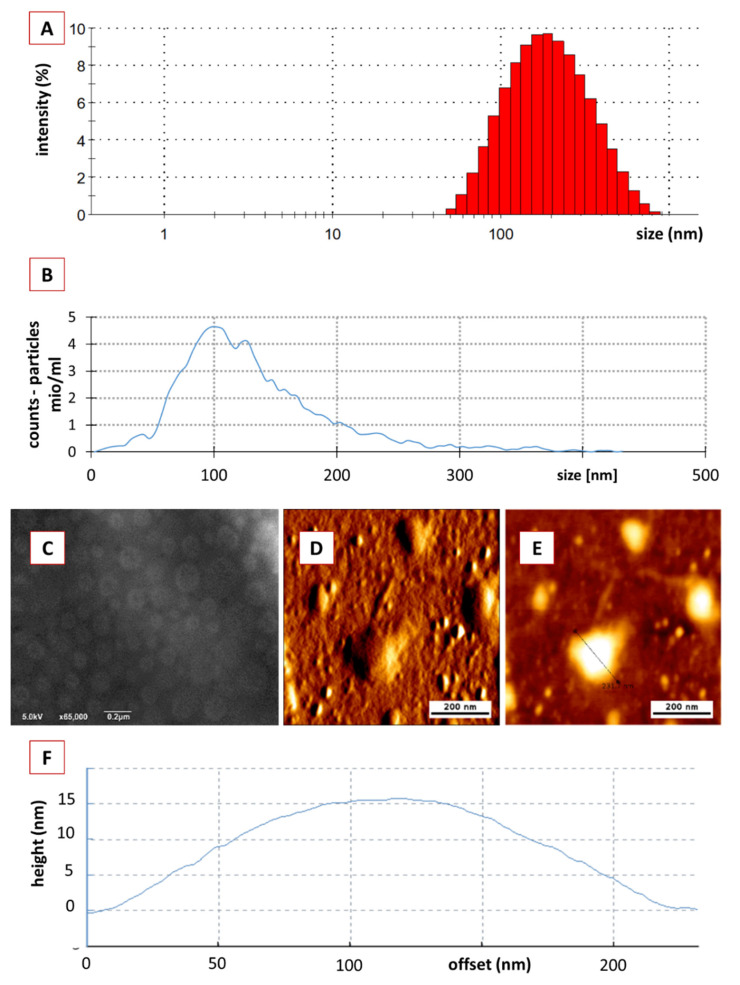
Physico-chemical characterization of crude classical PEVs. (**A**): DLS data, (**B**): NTA data, (**C**): SEM analysis, (**D**–**F**): AFM analysis.

**Figure 3:**
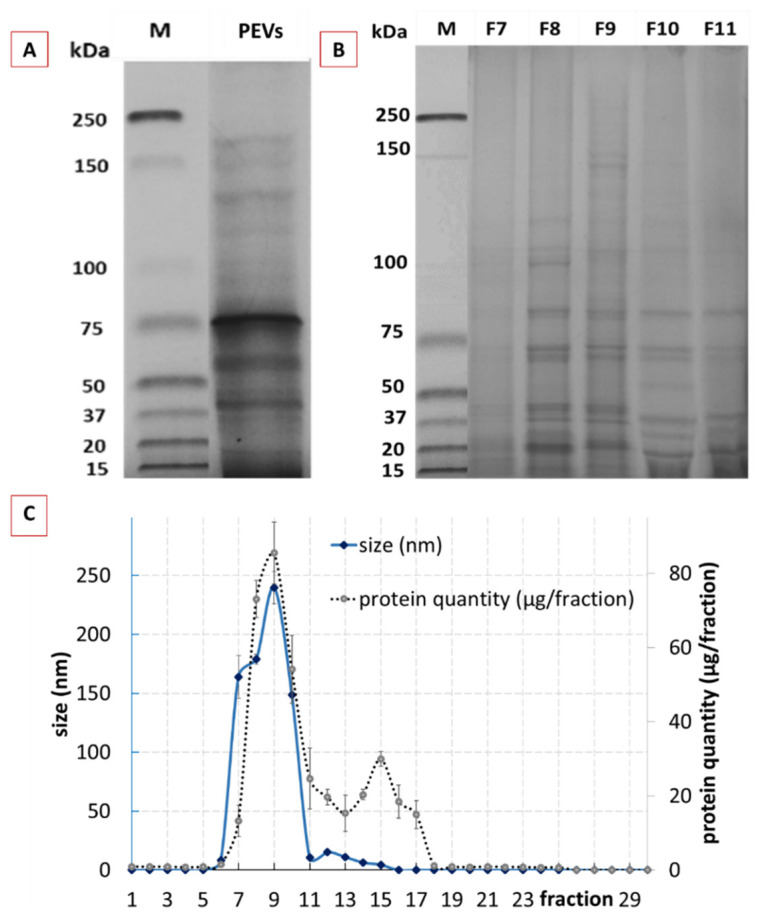
Physico-chemical characterization of crude and purified classical PEVs. (**A**): protein profile of crude PEVs, (**B**): protein profile of purified PEVs (selected fractions F7–F11), (**C**): DLS data and protein content of the 30 different fractions of purified classical PEVs.

The hydrodynamic diameter of the crude PEVs was 167± 3 nm and the polydispersity index (PdI) was 0.2 ± 0.02 (Figure 2A). NTA analysis determined a particle size of 123 nm, showed a relatively broad size distribution (<40 nm–>400 nm) and a particle concentration of 1.0 × 10^12^ particles/mL (Figure 2B), which is well in agreement with previously reported data for the size and size distribution of crude EVs [55,56,57]. The ZP of the crude PEVs was −32 ± 0.13 mV, indicating good physical stabilization of the PEVs by electrostatic stabilization [30,58]. SEM and AFM analysis (Figure 2C–E) confirmed the size and size distribution results obtained from DLS and NTA and revealed imperfect spherical shaped PEVs with inhomogeneous surface, which can be explained by the presence of proteins inside the highly dense lipid membrane [59,60]. AFM was also used to analyse the height of the PEVs, which was found to be 15 nm (Figure 2F). Hence, the PEVs appeared rather flat and not spherical. The flattening of the PEVs is considered to occur during the drying step prior to the AFM analysis. This means that the low height can be considered an artefact, while the non-dried crude PEVs are most likely spherical in shape [60,61]. The protein content within the crude PEVs was 6.22 ± 0.83 µg protein/g of cucumber and the protein profile showed numerous bands throughout the gel (Figure 3A), as it is usually noticed in EVs [62].

#### 3.1.2. Characterization of Purified Classical PEVs

Purification of the crude classical PEVs resulted in 30 fractions with different sizes, protein content and profile (Figure 3B,C). The size profile of the fractionated crude PEVs showed one peak with sizes >100 nm in between the fractions 7–10 (Figure 3C—blue line), indicating that these fractions contained the cucumber-derived PEVs [24]. The total protein yield after purification was 76% and the protein elution profile resulted in two broad peaks (Figure 3C—dotted line). The first peak corresponded to the PEVs size fraction (F8–F10) and the second peak was found within the fractions F11–F18. The bimodal protein elution profile obtained is in line with a study published for tomato juice and, therefore, indicates that the first peak corresponds to the PEVs (F8–F10), whereas the second peak is derived from soluble proteins, which were late eluted as they were entrapped in the pores of the used beads [24]. The elution profile also shows that most of the proteins are entrapped in the PEVs and that only minor proportions of the protein were not entrapped within the PEVs. Hence, as already shown in previous studies, SEC proved to be an efficient method for the purification of PEVs.

**Figure 4:**
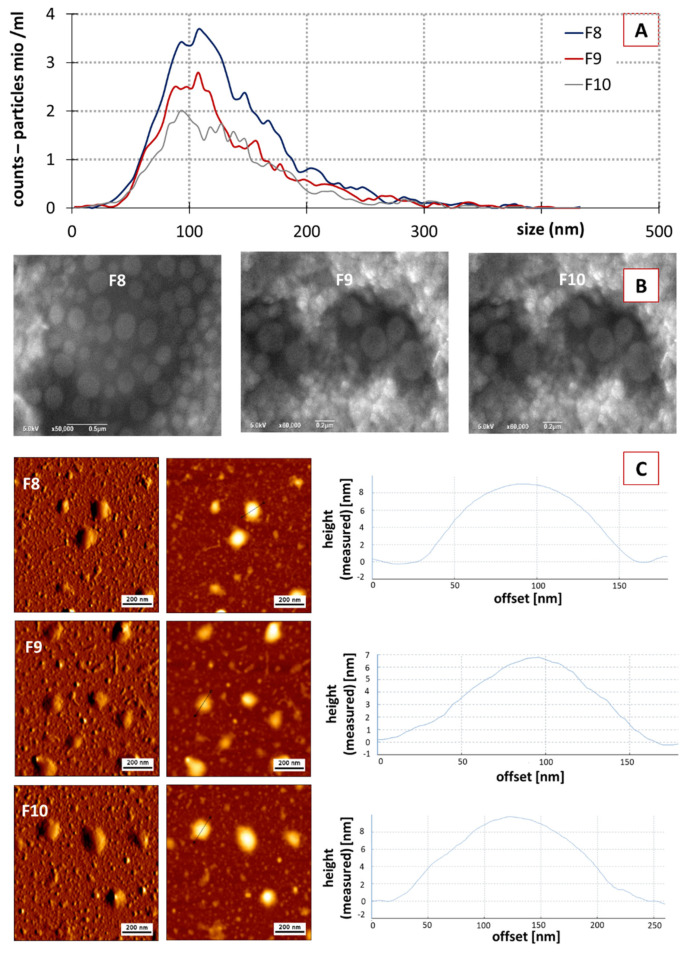
Physico-chemical characterization of fractions F8–F10 from purified classical PEVs. (**A**): NTA analysis, (**B**): SEM analysis, (**C**): AFM analysis.

In order to prove that F8–F10 corresponded, indeed, to the purified PEVs; these fractions were further characterized by using NTA, SEM, AFM and ZP analysis (Figure 4). The protein profiles were also determined (Figure 3B). The protein profile of F8, F9 and F10 showed less bands in comparison to the crude PEVs (Figure 3B), indicating that the purified PEVs possess less protein complexity when compared to the crude PEVs. F8 and F9 represented a very identical protein profile and F10 showed a similar profile with slight differences in the protein abundances when compared to the previous two fractions. In addition to F8–F10, the protein profiles of F7 and F11 were also determined (Figure 3B). F7 showed almost no protein bands and F11 far fewer and less clearly visible bands. Results, therefore, prove that only the fractions F8–F10 contain PEVs.

No significant differences in size and size distribution were found between the different PEVs fractions in the NTA analysis (Figure 4A). For all fractions, the mean particle size was in the range between 119–124 nm; all fractions possessed a very broad size distribution being similar to the size distribution of the crude PEVs (cf. Figure 2B). 

SEC resulted in a reduced particle concentration. F8 showed a particle concentration of 3.8 × 10^10^ particle/mL, followed by F9 and F10 with values of 5.2 × 10^9^ and 4.1 × 10^9^, respectively (Figure 4A). ZP values of the purified fractions were similar to the crude PEVs, with values being −37 ± 0.3, −35 ± 0.1 and −32 ± 0.08 mV for F8, F9 and F10, respectively. Hence, the purified PEVs can also be considered to possess excellent physical stability due to electro-static stabilization [30,58]. SEM and AFM analysis also confirmed the presence of the PEVs in F8, F9 and F10, with similar properties when compared to the crude PEVs (Figure 4B,C). Data, therefore, demonstrate that it was possible to obtain PEVs from cucumber via classical isolation and purification procedures, i.e., DUC and SEC. The cucumber-derived PEVs possess similar properties as those that were already reported for other PEVs.

### 3.2. Cucumber-Derived PEVs Produced by PlantCrystal-Technology (HPH)

#### 3.2.1. Characterization of Crude PlantCrystal-PEVs

**Figure 5:**
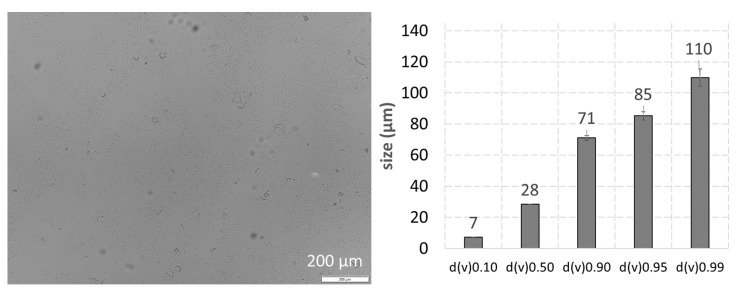
Physico-chemical characterization of crude PlantCrystal-PEVs—(**left**): light microscopy, (**right**): LD analysis.

High pressure homogenization of cucumber resulted in a mean particle size of >4000 nm and a PdI > 0.8 (DLS data). With this large size and the broad size distribution, the size of the cucumber PlantCrystals cannot be reliably assessed via DLS and NTA. Hence, other techniques were required to obtain meaningful size results for the cucumber PlantCrystals. Therefore, LD and LM were utilized for the size characterization of the cucumber PlantCrystals (Figure 5).

LM and LD showed that the cucumber PlantCrystals were composed of a mixture of differently sized particles. The mixture contained submicron particles but also larger-sized fiber-like particles in the micrometer range (Figure 5), indicating that not all parts of the plants could be successfully destroyed by HPH. The ZP of the crude PlantCrystal-PEVs was −22 ± 2 mV. Data indicate that the PEVs and the other particles in the PlantCrystal suspension possess a different particle surface than the classical PEVs. The lower ZP of the PlantCrystal-PEVs, when compared to the classical PEVs, suggests an electro-statical stabilization of the PlantCrystal-PEVs. However, the lower value indicates a less sufficient physical stability when compared to the classical PEVs.

#### 3.2.2. Characterization of Purified PlantCrystal-PEVs

The next step aimed at purifying the crude PlantCrystal-PEVs and to obtain PEV-containing fractions without the larger sized particles that could be used for a more detailed determination of the physico-chemical properties of the PlantCrystal-PEVs. Unexpectedly, it was not possible to obtain different fractions from the cucumber PlantCrystals via SEC; in addition, GU was not able to obtain the different layers that are typically obtained in EVs purification using GU. Instead, only one cloudy fraction over all the tube was noticed. Hence, results showed that the classical EVs purification procedures are not suitable to obtain purified PlantCrystal-PEVs. More research is, therefore, needed to identify methods that enable a successful purification of PlantCrystal-PEVs from crude PlantCrystal suspensions. As a purification of PlantCrystal-PEVs and their subsequent characterization was not possible in this study, a direct proof as to whether HPH can be used for the production of PlantCrystal-PEVs was not possible with this set of data. Therefore, the next part of the study aimed at providing an indirect proof or falsification for the existence of PlantCrystal-PEVs upon HPH. By assuming that PEVs are formed during HPH, it was hypothesized that the addition of an AI-surrogate to the cucumber suspension prior to the HPH should lead to the encapsulation of the AI-surrogate into the PEVs that are formed during the HPH. In contrast, it was hypothesized that the addition of the AI-surrogate to the cucumber suspension after HPH would not allow for an effective encapsulation of the AI-surrogate into the PEVs, because the PEVs are expected to be formed during HPH. Hence, the addition of AI-surrogate after HPH is considered to cause a distribution of the AI-surrogate outside the PEVs. In case no PEVs are formed during HPH, the AI-surrogate should be distributed similarly in both formulations; hence, no differences in the biopharmaceutical efficacy can be expected.

In order to prove this theory, AI-surrogate-loaded PlantCrystals were produced. The AI-surrogate was added to the “loaded PlantCrystal-PEVs” prior to the HPH and to the “PlantCrystal-PEVs + DiI” after HPH (cf. Table 1). Loading of DiI to the cucumber PlantCrystals caused no changes in the size and the size distribution of the particles (cf. Supplementary Section S2). However, small changes were found for the ZP (Table 2). The highest ZP was found for the non-loaded PlantCrystals and the lowest ZP was found for the PlantCrystals to which the AI-surrogate was added after HPH. The observed decrease in ZP after the addition of the AI-surrogate is reasonable because the AI-surrogate used in this study possesses a positive charge, which reduces the negative ZP of the PlantCrystals. The reduction in ZP was less pronounced when the AI-surrogate was added to the cucumber suspension prior to HPH. This indicates that parts of the AI-surrogate might have been encapsulated into PEVs during HPH. This results in less free positively charged AI-surrogate outside the PEVs and, thus, causes a less pronounced reduction in the ZP.

### 3.3. Dermal Penetration Efficacy of AI Surrogate-Loaded PEVs

**Figure 6:**
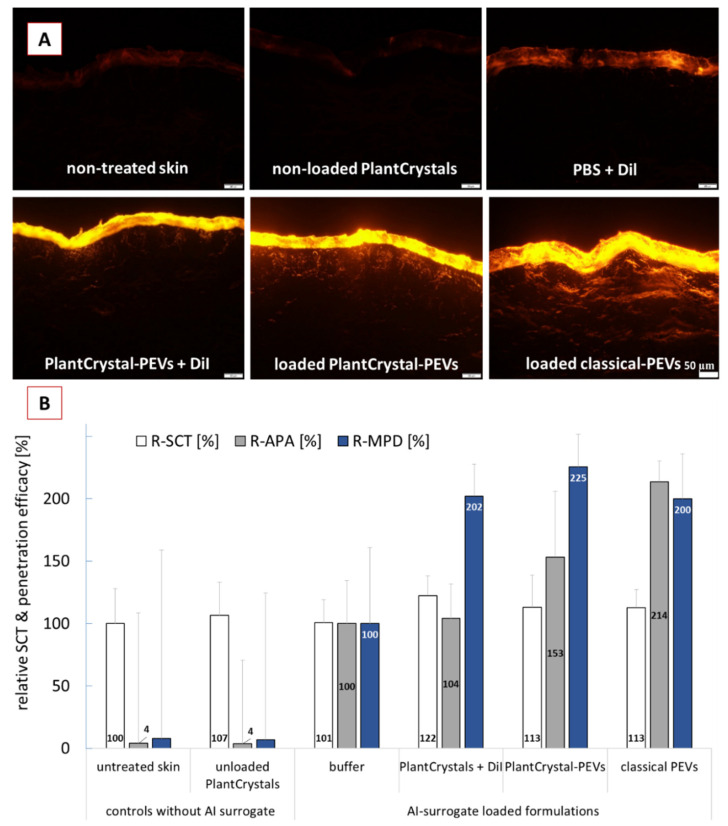
Dermal penetration efficacy of DiI used as AI surrogate in the different formulations. (**A**): images from skin sections obtained by inverted epifluorescence microscopy (all images taken with identical settings: 200-fold magnification, 50 ms exposure time, 100% attenuator of light source set to 100%, scale bar = 50 µm), (**B**): penetration parameters obtained with digital image analysis (R-SCT = relative stratum corneum thickness, R-APA = relative amount of penetrated AI-surrogate, R-MPD = relative mean penetration depth of AI surrogate).

In the next step, the different formulations were applied on skin and the dermal penetration efficacy was determined (Figure 6). The images of the skin sections obtained from inverted epifluorescence microscopy showed pronounced differences for the penetration efficacy of the AI-surrogate from the different formulations (Figure 6A); the results from digital image analysis confirmed these observations (Figure 6B).

The least penetration (lowest MPD and lowest APA) was found when the AI-surrogate DiI was added to PBS. The addition of DiI to the PlantCrystals after HPH resulted in a significant increase in the MPD (about 2-fold) but could not increase the APA. Addition of the DiI to the cucumber suspension prior to the HPH (PlantCrystal-PEVs) also resulted in a roughly 2-fold higher MPD and led to an increase in APA by about 50%. The application of classical PEVs also caused a roughly 2-fold higher MPD and 2-fold higher APA when compared to the DiI applied in buffer (Figure 6B). Data show that the cucumber-derived PEVs that were obtained by the classical preparation methods for EVs can improve the dermal penetration efficacy of the AI-surrogate. With this they provide further evidence and confirm that PEVs are suitable nanocarriers for improved (dermal/transdermal) drug delivery.

Moreover, data can also provide an indirect proof that HPH is able to form PEVs in which the AI-surrogate is encapsulated. The data, therefore, support our theory and substantiate the findings from the ZP measurements. Moreover, the SCT measurements indicate that the improvement in penetration efficacy was due to the encapsulation of the DiI in PEVs and was not related to differences in the skin hydration. Skin hydration is known to promote the dermal penetration. The PlantCrystals to which the AI-surrogate was added after HPH caused a significant increase in SCT by about 22% (Figure 6B). This resulted in an increased MPD for the AI surrogate but caused no increase in APA. The application of loaded classical PEVs and loaded PlantCrystal-PEVs caused an increase in SCT by about 13%. The increase in SCT was significant when compared to the SCT, where DiI was applied in buffer. However, it was not significantly different to the SCT after application of the PlantCrystals to which DiI was added after the HPH. Hence, the hydration of the SC was similar for all formulations that contained DiI and cucumber; thus, the hydration of the SC cannot be considered to cause the different APA values. Consequently, PEVs can be considered to be the cause for the improved APA values. By considering a 2-fold increase in APA for the classical PEVs, a 1.5-fold increase in APA for the PlantCrystal-PEVs and no increase for the PlantCrystals to which DiI was added after HPH, it can be speculated that about 100% of the AI-surrogate was encapsulated in the classical PEVs and about 50% of the AI-surrogate seemed to be encapsulated in the PlantCrystal-PEVs. In contrast, no encapsulation of the AI-surrogate was expected for the PlantCrystals, to which DiI was added after HPH. 

Further research is now needed to gain more detailed information on the structures and encapsulation efficacy of AI into the differently produced PEVs and PlantCrystal formulations. However, data obtained from this study already allow us to conclude that HPH can be used to produce PEVs that allow for an improved dermal drug delivery. Data also proved that classical methods for the preparation of PEVs can also be used to obtain cucumber-derived PEVs that allow for improved dermal drug delivery. 

## 4. Conclusions

Cucumber-derived PEVs were successfully obtained and characterized with classical methods typically used for EVs isolation and characterization. The cucumber-derived PEVs were demonstrated to increase the dermal penetration efficacy of a lipophilic AI-surrogate to about 200%. In addition to the classical PEVs, PEVs were also produced by HPH. The presence of PEVs after HPH could not be demonstrated with classical characterization methods. However, an indirect proof was achieved by demonstrating an increase in penetration efficacy for the AI surrogate after HPH, which could not be obtained without HPH. Further research is now needed to gain more detailed knowledge on the structure and the composition of HPH-PEVs. The influence of the production parameters (homogenization pressure, number of homogenization cycles) on the structure and the biopharmaceutical performance of the HPH-PEVs should also be investigated to allow for a production of more effective HPH-PEVs in the future. Ultimately, the present study showed that HPH is a promising technique for the production of PEVs that can be used for improved drug delivery.

## Figures and Tables

**Table 1:** Overview of formulations and controls tested on the ex-vivo porcine ear model.

Sample Name	Composition	AI Surrogate = DiI (10 nM)
• **Controls**		
blank (untreated skin)	-	−
PlantCrystal-PEVs	HPH processed cucumber juice without DiI	−
DiI-PBS	DiI added into PBS	+
PlantCrystal-PEVs + DiI	DiI added into HPH processed cucumber juice	+
• **AI-Surrogate-Loaded PEVs**		
loaded classical-PEVs	DiI incorporated into classical PEVs	+
loaded PlantCrystal-PEVs	DiI incorporated into PlantCrystal-PEVs	+

**Table 2:** Zeta potentials of cucumber PlantCrystals with and without AI-surrogate.

Sample	ZP [mV] ± SD
non loaded cucumber PlantCrystals	−22 ± 2
loaded PlantCrystal-PEVs *	−18 ± 2
PlantCrystal-PEVs + DiI **	−16 ± 2

* AI surrogate was added to the cucumber suspension prior to HPH, ** AI surrogate was added to the cucumber suspension after HPH.

## Data Availability

All data generated or analyzed during this study are included in this published article and Supplementary Materials.

## Supplementary Materials

The following supporting information can be downloaded at: https://www.mdpi.com/article/10.3390/pharmaceutics14030476/s1, S1: ImageJ macro. S2: Figure S1: Physico-chemical characterization of the DiI-loaded PlantCrystal formulations—LD data.; Figure S2: Physico-chemical characterization of the DiI-loaded PlantCrystal formulations—DLS data.; Figure S3: Physico-chemical characterization of the DiI-loaded PlantCrystal formulations—Light microscopy analysis. A= cucumber + HPH, B = cucumber + HPH + DiI, C= cucumber + DiI + HPH.
