# Supplementary material for: Cucumber-Derived Exosome-like Vesicles and PlantCrystals for Improved Dermal Drug Delivery
Source: Pharmaceutics. 2022 Feb 22;14(3):476. doi: 10.3390/pharmaceutics14030476 (PMC8955785; doi:10.3390/pharmaceutics14030476)
Supplement: Supplementary file 1 [file pharmaceutics-14-00476-s001.zip › pharmaceutics-1596204-supplementary.pdf]

pharmaceutics

Supplementary materials Cucumber-derived Exosome-like Vesicles and PlantCrystals for Improved Dermal Drug Delivery

Abraham M. Abraham, Sabrina Wiemann, Ghazala Ambreen, Jenny Zhou, Konrad Engelhardt, Jana Brüßler, Udo Bakowsky, Shu-Ming Li, Robert Mandic, Gabriella Pocsfalvi and Cornelia M. Keck *

Section 1.

The following ImageJ macro was recorded and used to eliminate the autofluorescence of the skin for all images obtained from inverted epifluorescence microscopy:

```
run("Convert Stack to Images");
selectWindow("Red");
rename("0");
selectWindow("Green");
rename("1");
selectWindow("Blue");
rename("2");
min[0]=0;
max[0]=0;
filter[0]="stop";
min[1]=20;
max[1]=255;
filter[1]="pass";
min[2]=0;
max[2]=0;
filter[2]="stop";
for (i=0;i<3;i++){
selectWindow(""+i);
setThreshold(min[i], max[i]);
run("Convert to Mask");
if (filter[i]=="stop")  run("Invert");
}
imageCalculator("AND create", "0","1");
imageCalculator("AND create", "Result of 0","2");
for (i=0;i<3;i++){
selectWindow(""+i);
close();
}
selectWindow("Result of 0");
close();
selectWindow("Result of Result of 0");
rename(a);
// Colour Thresholding-------------
run „invert“
```

Section 2.

Physico-chemical characterization of the DiI-loaded PlantCrystal formulations. Figure S1 – LD data, Fig. S2 – DLS data, Fig. S3 – Light microscopy data.

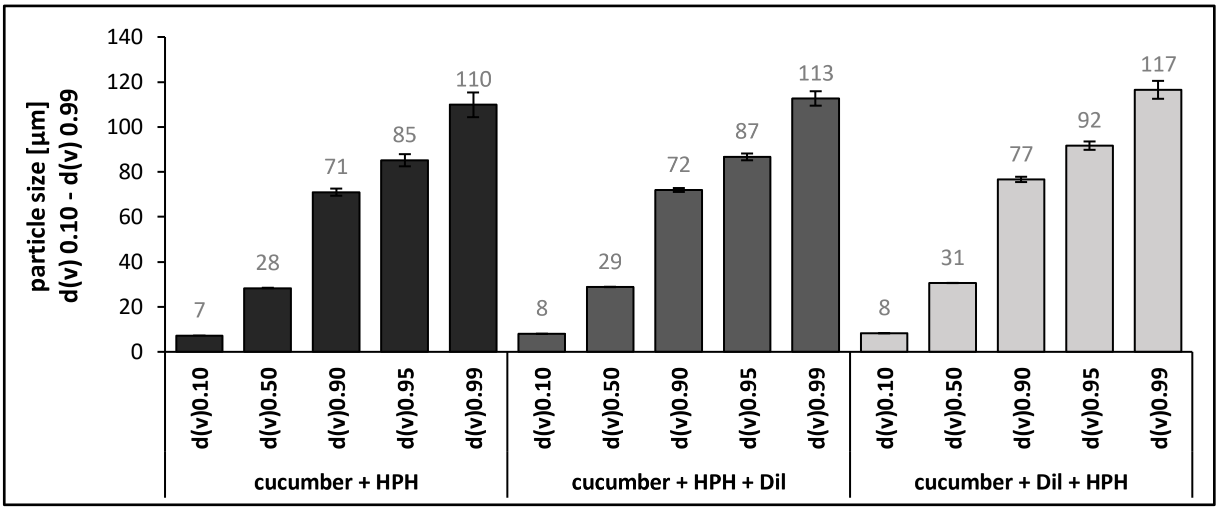

Figure S1. Physico-chemical characterization of the DiI-loaded PlantCrystal formulations - LD data.

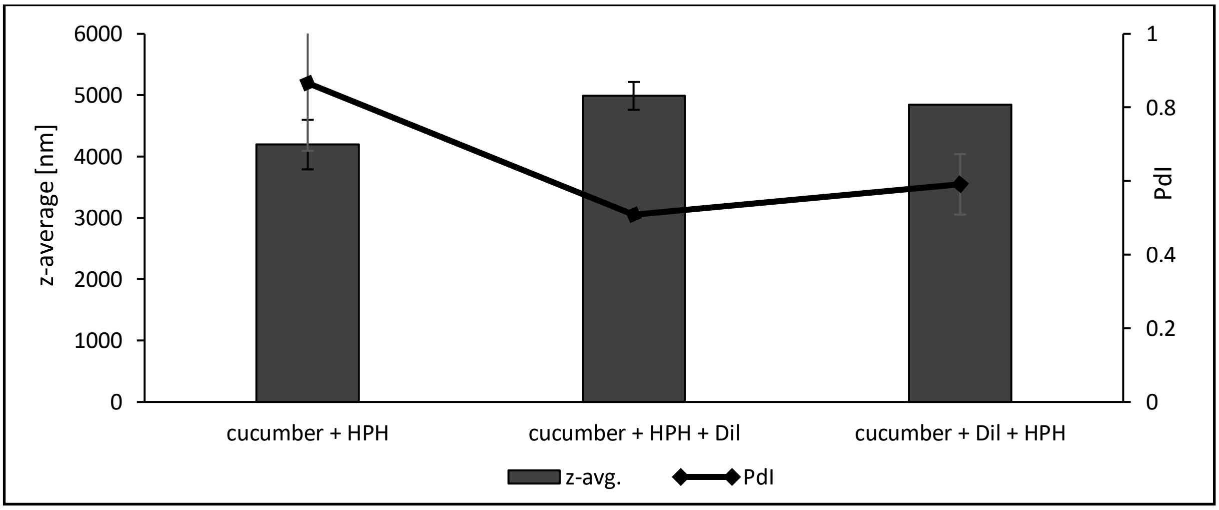

Figure S2. Physico-chemical characterization of the DiI-loaded PlantCrystal formulations – DLS data.

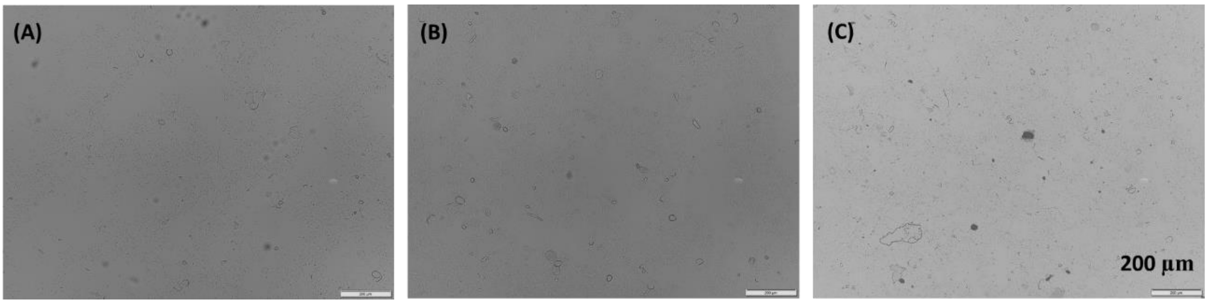

Figure S3. Physico-chemical characterization of the DiI-loaded PlantCrystal formulations – Light microscopy analysis. A= cucumber + HPH, B = cucumber + HPH + DiI, C= cucumber + DiI + HPH.
